# Prayer-for-health and complementary alternative medicine use among Malaysian breast cancer patients during chemotherapy

**DOI:** 10.1186/1472-6882-14-425

**Published:** 2014-10-30

**Authors:** Ping Lei Chui, Khatijah Lim Abdullah, Li Ping Wong, Nur Aishah Taib

**Affiliations:** Department of Nursing Science, Faculty of Medicine, University of Malaya, Kuala Lumpur, 50603 Malaysia; Department of Social and Preventive Medicine, Faculty of Medicine, University of Malaya, Kuala Lumpur, 50603 Malaysia; Department of Surgery, Faculty of Medicine, University of Malaya, Kuala Lumpur, 50603 Malaysia

**Keywords:** Complementary alternative therapy, Prayer for health, Breast cancer, Chemotherapy

## Abstract

**Background:**

The inclusion of prayer-for-health (PFH) in the definition of complementary alternative medicine (CAM) has resulted in higher levels of CAM use. The objective of this study was to assess PFH and CAM use among breast cancer patients undergoing chemotherapy.

**Methods:**

A cross-sectional study was performed at two chemotherapy providers. Patients were questioned about use of three categories of CAM, mind-body practices (MBPs), natural products (NPs) and traditional medicine (TM). PFH was also examined separately from CAM to better characterise the patterns of CAM and PFH used during chemotherapy.

**Results:**

A total of 546 eligible patients participated in the study; 70.7% (n = 386) reported using some form of CAM, and 29.3% (n = 160) were non-CAM users. When PFH was excluded as a CAM, fewer patients reported the use of CAM (66.1%; n = 361). The total number of patients who used MBPs decreased from 342 to 183. The most common CAM use category was NPs (82.8%), followed by MBPs (50.7%), and TM (35.7%). CAM users were more likely to have a tertiary education (OR 2.11, 95% CI 1.15–3.89 vs. primary/lower), have household incomes > RM 3,000 (≈944 USD) per month (OR 2.32, 95% CI 1.40–3.84 vs. ≤RM 3,000 (≈944 USD)), and have advanced cancer (OR 1.75, 95% CI 1.18–2.59 vs. early stage cancer), compared with non-CAM users. The CAM users were less likely to have their chemotherapy on schedule (OR 0.24, 95% CI 0.10–0.58 vs. chemotherapy postponed) than non-CAM users. Most MBPs were perceived to be more helpful by their users, compared with the users of NPs and TM.

**Conclusion:**

CAM use was prevalent among breast cancer patients. Excluding PFH from the definition of CAM reduced the prevalence of overall CAM use. Overall, CAM use was associated with higher education levels and household incomes, advanced cancer and lower chemotherapy schedule compliance. Many patients perceived MBP to be beneficial for improving overall well-being during chemotherapy. These findings, while preliminary, clearly indicate the differences in CAM use when PFH is included in, and excluded from, the definition of CAM.

**Electronic supplementary material:**

The online version of this article (doi:10.1186/1472-6882-14-425) contains supplementary material, which is available to authorized users.

## Background

The use of complementary alternative medicine (CAM) has become increasingly popular [1], particularly among cancer patients [2, 3]. The average prevalence of CAM use among cancer patients in Western countries is 40% [4]. The prevalence of CAM use among cancer patients in Asia is 55.0% [5] and 56.0% [6] in Singapore, 60.9% [7] in Thailand, 36.0% [8] and 71.5% [9] in Turkey, 97.0% [10] in China, 57.4% [11] in Korea, 79.3% [12] and 98.1% [13] in Taiwan, 56.6% [14] in India, and 59.0% [15] in Brunei Darussalam. The prevalence of CAM use by cancer patients varies by population, study design and by different study definitions of CAM [16].

In Malaysia, the term traditional and complementary alternative medicine (TM&CAM) is used to denote health-related practices that are not provided by registered conventional medical practitioners to prevent, treat and/or manage illness, and/or preserve the mental and physical well-being of individuals [17]. In general, the term TM&CAM is used inter-changeably with the term CAM. Siti et al. reported that the prevalence of TM&CAM ever used in a lifetime among Malaysians was 69.4% (67.6–71.2%) and in the last 12-month period was 55.6% (53.8–57.4%) [18]. The prevalence of CAM use by breast cancer patients ranges from 51.0 to 88.3% [19–21]. The CAM practised in Malaysia reflects the diverse population of Malay, Chinese, Indian and indigenous cultures. Ethnic Malays represent the majority of the population (67.4%), followed by Chinese (24.6%), Indian (7.3%) and other local (0.7%) ethnic populations. Approximately 61.3% of the population practices Islam, 19.8% Buddhism, 9.2% Christianity, 6.3% Hinduism, and 2.6% practice Confucianism and other traditional Chinese religions [22]. The religion practiced by 1.0% of the population is unknown, 0.7% practice no religion and 0.4% practice an “other” religion. Traditional Malay/indigenous medical practices include healing techniques using natural resources, wafak (written symbols), and Quranic verses, supplication and offering of blessings to the Prophet Muhammad (PBUH) [23]. Depending on the healing techniques used, the Malay traditional healers are known as “bomoh” or Islamic healers. Traditional Chinese medicine consists of herbal medicines and other forms of treatment, including acupuncture, massage (Tui na), exercise (qigong) and dietary therapy [24]. The Chinese medical practitioners are known as *sinseh*. The traditional Indian medicines practised in Malaysia are Siddha, ayurveda and unani. Most of the medicines used are of vegetable, mineral or animal origin. These herbal preparations and products are imported from India as tablets, oils, ointments, metals, mineral concoctions and herbal powders [25].

In addition to traditional medicine, other commonly used CAM can be broadly categorised into mind-body practices (MBPs) and natural products (NPs) [24]. The NPs include all supplements ingested by participants. MBPs refer to all non-pharmacological modalities and include a large and diverse group of procedures or techniques administered, or taught, by a trained practitioner. In Malaysia, prayer for health (PFH) is included as an MBP because it is often used as a CAM therapy to aid in healing the body’s inner strength and reduce stress [18–20, 26, 27]. However, inclusion of PFH as a CAM therapy potentially inflates the number of reported CAM users [28, 29]. Therefore, PFH was examined separately from other MBPs to effectively address CAM use among the diverse populations in Malaysia. The objective of this study was to assess PFH and CAM use among breast cancer patients undergoing chemotherapy. Specifically, we investigated the characteristics associated with CAM use, patterns of CAM use, and users’ perceptions of the usefulness of, and reasons to use, CAM.

## Methods

### Design and setting

A cross-sectional survey was conducted at chemotherapy day-care centres at the Hospital Kuala Lumpur (HKL) and the University of Malaya Medical Centre (UMMC) in Malaysia. HKL is a government tertiary referral hospital with 2,302 beds. It is the principal Ministry of Health hospital in Malaysia and is one of the largest hospitals in Asia. UMMC is a renowned Ministry of Education teaching hospital, and has 980 beds. Both hospitals are referral centres for cancer care and provide day-care chemotherapy services to cancer patients from various parts of the country.

### Population and sampling

The average annual population of new breast cancer patients in both chemotherapy day-care centres is 605 patients per year. Hence, for an accuracy level of 0.95 with a margin of error ±2.0% and an expected prevalence of CAM use of 50%, the estimated sample size was 512 patients. An additional 10% of the calculated sample size was added to anticipate loss as a result of non-response and missing values. The final sample size was 563 participants. Participants were selected based on the following criteria: breast cancer patients who had undergone at least one cycle of chemotherapy and were waiting for their subsequent chemotherapy infusion (in any of two to six cycles of treatment), no previous history of cancer or previous chemotherapy, mentally and physically competent to participate in the study, and able to communicate in English, Bahasa Malaysia (Malay language), Mandarin or Tamil.

### Questionnaires

The questionnaires were developed based on questionnaires used in previous CAM studies [16, 20, 30] and on information from a literature review. The questionnaire consisted of three parts. Part I comprised items soliciting demographic, socioeconomic, disease and treatment characteristics. Part II consisted of multiple response items for CAM use, MBP (e.g., exercise, massage), PFH (self-performed prayer for own health/have asked others to pray for your health/participated in a prayer group with ritual or sacrament for your health), NP (e.g., vitamin and mineral supplements, cleansing and detoxifying diets, antioxidants), and TM (indigenous medicine). Twenty-seven types of CAM relevant to the local context were included, and dichotomous answer choices were used to elicit all therapies received by the patients. Open-ended responses were optional, and allowed participants to report any other CAM practices used that were not listed in the response choices. Participants were also asked to indicate the perceived helpfulness of the CAM used based on a 5-point Likert scale: (1 = not at all helpful, 2 = somewhat unhelpful, 3 = neither, 4 = somewhat helpful and 5 = very helpful). In Part III, an open-ended question was used to ask participants about their reasons for using CAM during the course of chemotherapy, “What do you think about the reasons for your CAM use?”. The open-ended questions were designed to encourage a full and meaningful answer. In total, the CAM use questionnaire consisted of 10 items that assessed demographic, socioeconomic, disease and treatment characteristics, 27 items and an open-ended question used to elicit all CAM therapies used by the patients, 27 items and an open-ended question to indicate the perceived helpfulness of the CAM practice used and an open-ended item used to explore the patients’ reasons for using CAM (Additional file 1).

Because the target population was multi-lingual and multi-ethnic, the questionnaires were available in English, Bahasa Malaysia, Mandarin and Tamil. Forward and backward questionnaire translations were performed to ensure semantic equivalence across languages and cultures. A panel of experts comprising oncology nurses, breast care nurses, and breast surgical oncologists performed the content validity testing. A convenience sample of 40 breast cancer patients undergoing chemotherapy from the chemotherapy day-care was included in the pilot study to confirm that the survey methods and instruments used were applicable and feasible. Ambiguous terms were replaced with the simpler and/or more common terms used by the nurses and patients. The questionnaire was then content validated by a panel of experts, and was finalised.

### Data collection

**Figure 1:**
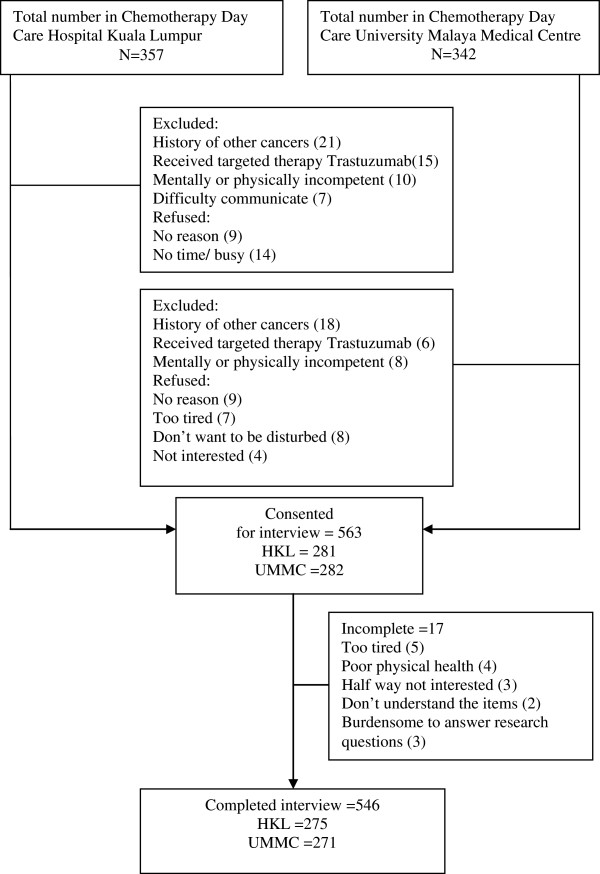
**Illustration of the inclusion process of the survey participants.**

Because the sample frame was small, all eligible patients who attended the UMMC and HKL chemotherapy day-care centres between March 2012 and August 2013 were approached and were informed about the study. Patients were excluded from the study if they had been diagnosed with a history of other cancers besides breast cancer, had previously received chemotherapy, received targeted therapy with trastuzumab instead of a chemotherapy regime, were mentally or physically incompetent, or were unable to communicate in English, Bahasa Malaysia, Mandarin or Tamil. Five hundred and sixty-three eligible patients consented to participate in the study. They were interviewed by the researcher in the health education room at the chemotherapy day-care centres. The participants were asked about the CAM they had used, particularly during chemotherapy, on their cancer- and chemotherapy-related side effects and symptoms. Each face-to-face structured interview was between 20 and 30 min in length. In total, 546 patients (97.0% response rate) completed the interview. The reasons for non-completion included being too tired, having poor physical health, lack of interest and answering similar questions in another study (Figure 1). The study was registered with the National Medical Research Registry (NMRR-10-111-5204) and was approved by the Ethical Committee of the Medical Research Ethnics Committee (MREC), Ministry of Health Malaysia, and the UMMC Medical Ethics Committee (Ref.770.18). All participants were reassured that confidentiality of their information would be maintained, and informed written consent was obtained from each participant.

### Statistical methods

The data were analysed using the Statistical Package for the Social Sciences (SPSS Ver. 18; IBM Corporation, Armonk, NY, USA). The dataset was examined to detect and correct inaccurate entries. Participants were classified as CAM users if they had used at least one type of CAM during the course of chemotherapy. PFH was examined separately from the CAM to better characterise the pattern of CAM and PFH used during chemotherapy. Descriptive statistics, frequencies and percentages were used to summarise the data. Associations that were significant in the univariate analysis were included in a multivariate logistic regression model to examine the associated characteristics that contributed to CAM use. A p-value <0.05 was considered statistically significant.

## Results

### Demographic, disease and treatment characteristics of the participants

A total of 546 patients participated in the study. Of these, 43.0% were in the 50- to 59-year age group, 45.1% were Malay, 53.3% had a secondary school education, 89.0% were married at some point and 72.0% had a household income ≤ RM 3,000 (≈944 USD) per month (i.e., low-income women) [31]. More than one-half (61.9%) were diagnosed with early-stage breast cancer, were post-menopausal (54.4%), were on FEC (5-fluorouracil (5-FU), epirubicin, and cyclophosphamide)/FAC (5-FU, doxorubicin and cyclophosphamide)/CMF (cyclophosphamide, methotrexate and 5FU)/AC (doxorubicin and cyclophosphamide) regimens (78.0%), were at chemotherapy cycles 2, 3 or 4 (67.4%), or had good compliance with the chemotherapy schedule (91.8%).

Some form of CAM was used by 70.7% (n = 386) of the patients, and 29.3% (n = 160) were non-CAM users. When PFH was excluded from the CAM, the number of patients that reported the use of some form of CAM decreased to 66.1% (n = 361). Despite some differences, the distributions of CAM users and non-CAM users (PFH included in CAM or PFH excluded from CAM) were very similar to the distributions of the overall participants. There were no significant differences in the proportions of CAM users by age, ethnicity, marital status, menopausal status, chemotherapy regimen or chemotherapy cycle. There were, however, significant differences (p < 0.05) in the proportions of CAM users by educational level, household incomes, disease stage and chemotherapy schedule adherence.

There were no significant differences in the proportions of PFH users by age, educational level, marital status, menopausal status, chemotherapy regimen or chemotherapy cycle and adherence. There were, however, significant differences (p < 0.001) between PFH users (61.6%) of Malay ethnicity, compared with non-PFH users (38.2%). PFH use was also significantly associated with household income (p < 0.001). More PFH users (39.6%) had household incomes > RM 3,000 (≈944 USD), compared with non-PFH users (23.3%). Use of PFH increased with disease stage (p < 0.001). Participants diagnosed with advanced-stage breast cancer (48.4%) were more likely to be PFH users, compared with non-PFH users (33.9%). The results for the demographic, socioeconomic, disease and treatment characteristics of CAM users and PFH users are presented in Table 1.

**Table 1:** **Characteristics of users and non-users of CAM and prayer-for-health**

		Included prayer-for-health			Excluded prayer-for-health			Prayer-for-health only		
Characteristics	All participants N = 546	CAM users N = 386	Non-CAM users N = 160	X ^2^	CAM users N = 361	Non-CAM users N = 185	X ^2^	Users N = 159	Non-users N = 387	X ^2^
	n (%)	n (%)	n (%)	***p***-value	n (%)	n (%)	***p***-value	n (%)	n (%)	
Age Mean ± SD 51.6 ± 9.2										
30–39	58 (10.6)	45 (11.7)	13 (8.1)	0.52	40 (11.1)	18 (9.7)	0.97	18 (11.3)	40 (10.3)	0.84
40–49	155 (28.4)	109 (28.2)	46 (28.8)		102 (28.3)	53 (28.6)		43 (27.0)	112 (28.9)	
50–59	235 (43.0)	167 (43.3)	68 (42.5)		154 (42.7)	81 (43.8)		72 (45.3)	163 (42.1)	
60 and above	98 (18.0)	65 (16.8)	33 (20.6)		65 (18.0)	33 (17.8)		26 (16.4)	72 (18.6)	
Ethnicity										
Malay	246 (45.1)	179 (46.4)	67 (41.9)	0.47	164 (45.4)	82 (44.3)	0.18	98 (61.6)	148 (38.2)	<.001**
Chinese	207 (37.9)	141 (36.5)	66 (41.2)		136 (37.7)	71 (38.4)		34 (21.4)	173 (44.7)	
Indian	80 (14.7)	54 (14.0)	26 (16.2)		49 (13.6)	31 (16.8)		24 (15.1)	56 (14.5)	
Others	13 (2.4)	12 (3.1)	1 (0.6)		12 (3.3)	1 (0.5)		3 (1.9)	10 (2.6)	
Education level										
Primary/lower	119 (21.8)	68 (17.6)	51 (31.9)	<.001**	65 (18.0)	54 (29.2)	<.001*	27 (17.0)	92 (23.8)	0.19
Secondary	291 (53.3)	203 (52.6)	88 (55.0)		188 (52.1)	103 (55.7)		88 (55.3)	203 (52.5)	
Tertiary	136 (24.9)	115 (29.8)	21 (13.1)		108 (29.9)	28 (15.1)		44 (27.7)	92 (23.8)	
Marital status										
Single	60 (11.0)	44 (11.4)	16 (10.0)	0.63	43 (11.9)	17 (9.2)	0.34	22 (13.8)	38 (9.8)	0.17
Ever married	486 (89.0)	342 (88.6)	144 (90.0)		318 (88.1)	168 (90.8)		137 (86.2)	349 (90.2)	
Household income/month										
≤RM 3000(≈944 USD)	393 (72.0)	253 (65.5)	140 (87.5)	<.001**	239 (66.2)	151 (83.2)	<.001*	96 (60.4)	297 (76.7)	<.001**
>RM 3000(≈944 USD)	153 (28.0)	133 (34.5)	20 (12.5)		122 (33.8)	31 (16.8)		63 (39.6)	90 (23.3)	
Staging of disease										
Early	338 (61.9)	228 (59.1)	110 (68.8)	0.03*	212 (58.7)	126 (68.1)	0.03*	82 (51.6)	256 (66.1)	<.001**
Advanced	208 (38.1)	158 (40.9)	50 (31.2)		149 (41.3)	59 (31.9)		77 (48.4)	131 (33.9)	
Menopausal status										
Pre menopause	249 (45.6)	184 (47.7)	65 (40.6|)	0.13	169 (46.8)	80 (43.2)	0.43	81 (50.9)	168 (43.4)	0.11
Post menopause	297 (54.4)	202(52.3)	95 (59.4)		192 (53.2)	105 (56.8)		78 (49.1)	219 (56.6)	
Chemotherapy regimen										
Docetaxel	120 (22.0)	91 (23.6)	29 (18.1)	0.16	86 (23.8)	34 (18.4)	0.15	37 (23.3)	83 (21.4)	0.64
FEC/FAC/CMF/AC	426 (78.0)	295 (76.4)	131 (81.9)		275 (76.2)	151 (81.6)		122 (76.7)	304 (78.6)	
Chemotherapy cycle										
2, 3 and 4	368 (67.4)	258 (66.8)	110 (68.8)	0.66	238 (65.9)	130 (70.3)	0.31	109 (68.6)	259 (66.9)	0.71
5 and 6	178 (32.6)	128 (33.2)	50 (31.2)		123 (34.1)	55 (29.7)		50 (31.4)	128 (33.1)	
Chemotherapy adherence										
Postponed	45 (8.2)	41 (10.6)	4 (2.5)	<.001**	39 (10.8)	6 (3.2)	<.001**	10 (6.3)	35 (9.0)	0.28
On schedule	501 (91.8)	345 (89.4)	156 (97.5)		322 (89.2)	179 (96.8)		149 (93.7)	352 (91.0)	

**p < 0.001.

*p < 0.05.

### Correlates of CAM use

The multivariate logistic regression analysis revealed that CAM users (PFH included in CAM; PFH excluded from CAM) were more likely to have a tertiary education (odds ratio (OR) 2.33, 95% confidence interval (CI) 1.22–4.47; OR 2.11, 95% CI 1.15–3.89 vs. primary school/lower), have average household incomes > RM 3,000 (≈944 USD) per month (OR 3.41, 95% CI 1.92–6.03; OR 2.32, 95% CI 1.40–3.84 vs. ≤RM 3,000 (≈944 USD)), with advanced-stage cancer (OR 1.86, 95% CI 1.23–2.82; OR 1.75, 95% CI 1.18–2.59 vs. early stage of cancer) than non-CAM users. The CAM users (PFH included in CAM; PFH excluded from CAM) were less likely to comply with the chemotherapy schedule (OR 0.18, 95% CI 0.06–0.51; OR 0.24, 95% CI 0.10–0.58 vs. chemotherapy postponed) compared with non-CAM users (Table 2).

**Table 2:** **Logistic regression analysis results for significant characteristics between users and non-users of CAM and prayer-for-health**

	Multivariate logistic regression model of		
	CAM users vs. non-CAM users† (Included prayer-for-health)	CAM users vs. non-CAM users‡ (Excluded prayer-for-health)	Users vs. non-users§ (Prayer-for-health only)
Characteristics	OR (95% CI)	OR (95% CI)	OR (95% CI)
Ethnicity			
Malay	-	-	Reference
Chinese			0.31 (0.20–0.49)**
Indian			0.81 (0.46–1.42)
Others			0.57 (0.15–2.18)
Education level			
Primary school/lower	Reference	Reference	-
Secondary school	1.44 (0.90–2.24)	1.28 (0.82–1.99)	
Tertiary	2.33 (1.22–4.47)*	2.11 (1.15–3.89)*	
Household income/month			
≤RM 3000 (≈944 USD)	Reference	Reference	Reference
>RM 3000 (≈944 USD)	3.41 (1.92–6.03)**	2.32 (1.40–3.84)**	2.55 (1.66–3.93)**
Staging of disease			
Early	Reference	Reference	Reference
Advanced	1.86 (1.23–2.82)**	1.75 (1.18–2.59)*	2.10 (1.39–3.13)**
Chemotherapy adherence			
Postponed	Reference	Reference	-
On schedule	0.18 (0.06–0.51)**	0.24 (0.10–0.58)**	

†Logistic regression model: Hosmer and Lemeshow test, χ^2^ = 3.47 (5), *p* = 0.63.

‡Logistic regression model: Hosmer and Lemeshow test, χ^2^ = 8.46 (6), *p* = 0.21.

§Logistic regression model: Hosmer and Lemeshow test, χ^2^ = 11.43 (6), *p* = 0.08.

**p < 0.001.

*p < 0.05.

### Comparison of CAM use (PFH included in definition of CAM vs. PFH excluded from the definition of CAM)

**Figure 2:**
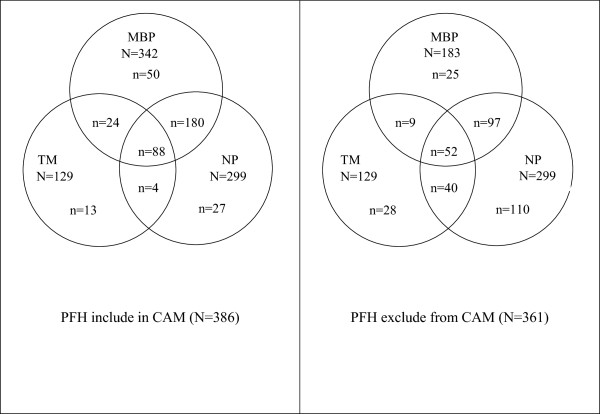
**Overlap in CAM use when prayer-for-health (PFH) is included in, and excluded from, CAM.**

When PFH was included in the definition of CAM, the most common CAM use was MBPs (88.6%), followed by NPs (77.5%), and TM (33.4%). When PFH was excluded from the CAM definition, the total number of patients who performed MBPs decreased from 88.6% (n = 342) to 50.7% (n = 183). The most common CAM use became NPs (82.8%), followed by MBPs (50.7%), and TM (35.7%). Three-quarters (76.7%, n = 296) of CAM users (PFH included in CAM) and over one-half (54.8%, n = 198) of CAM users (PFH excluded from CAM) used a combination of MBPs or/and NPs or/and TM. Figure 2 presents the results for the comparison between MBPs, NPs, and TM use when PFH was included in the definition of CAM and when PFH was excluded from the definition of CAM.

Most of the patients were users of multiple therapies. Almost one-half (46.5%) of the MBPs users (PFH included in CAM) and 39.3% of the MBPs users (PFH excluded from CAM) reported using more than one MBP therapy (Table 3). Greater than one-half (62.9%) of the NPs users ingested more than one NP during chemotherapy. Almost all (97.7%) of the TM users sought only a single TM therapy.

**Table 3:** **Numbers of therapies used by MBP, NP and TM users**

	MBP users (PFH included in CAM) N = 342	MBP users (PFH excluded from CAM) N = 183	NP users N = 299	TM users N = 129
	n (%)	n (%)	n (%)	n (%)
One therapy	183 (53.5)	111 (60.7)	111 (37.1)	126 (97.7)
Two therapies	91 (26.6)	47 (25.7)	109 (36.5)	3 (2.3)
Three therapies	43 (12.6)	7 (3.8)	52 (17.4)	0 (-)
More than three therapies	25 (7.3)	18 (9.8)	27 (9 .0)	0 (-)

### CAM use

PFH was performed by 310 CAM users. The most popular therapy in the MBP category was relaxation exercises (n = 67), followed by massages (n = 60), meditation (n = 52), tai chi (n = 33), yoga (n = 29) and therapeutic/healing touch (n = 24). Other MBPs were less common, and <10% of the MBP users engaged in these methods. The most popular NPs were vitamin and mineral supplements (n = 168), cleansing/detoxifying diets (n = 68), antioxidant capsules/tablets (n = 61), cactus juice (n = 47) and spirulina (n = 43). The other, less frequently used, NPs were used by <10% of the NP users. The open-ended questions revealed an additional nine NPs that were ingested by patients during chemotherapy (i.e., cactus juice, protein powder, immune booster, grape seed extract, mangosteen juice, porcupine date, *noni* juice, aloe vera, and enzymes). Most of the TM users visited a Malay traditional healer (n = 96). Twenty-four of the TM users received traditional Chinese medicine treatments. Less than 10% of the patients reported using homeopathy. None of the patients reported using ayurveda/siddha/Unani. Table 4 presents the results for the various CAMs used by the breast cancer patients during chemotherapy.

**Table 4:** **Type of CAM use and perceived helpfulness by CAM users during chemotherapy**

Type of CAM	N	Perceived helpfulness		
		Not helpful	Neither	Helpful
		n (%)		
Prayer-for-health	310	1 (0.3)	12 (3.9)	297 (95.9)
*Mind-Body Practices N = 183*				
Relaxation exercise	67	-	10 (14.9)	57 (85.1)
Massage	60	-	7 (11.7)	53 (88.3)
Meditation	52	2 (3.8)	6 (11.5)	44 (84.6)
Tai Chi	33	-	-	33 (100)
Yoga	29	-	3 (10.3)	26 (89.6)
Therapeutic/healing touch	24	2 (8.3)	8 (33.3)	14 (58.3)
Aromatherapy	17	-	1 (5.9)	16 (94.1)
Reiki	15	-	5 (33.3)	10 (66.7)
Cupping/bekam	4	-	-	4 (100)
Acupuncture	4	-	2 (50.0)	2 (50)
*Natural Products N = 299*				
Vitamin & mineral supplement	168	4 (2.4)	32 (19.0)	132 (78.6)
Cleansing detoxifying diet	68	6 (8.8)	13 (19.1)	49 (72.0)
Antioxidant capsule/tablet	61	4 (6.6)	20 (32.8)	37 (60.6)
Cactus juice†	47	-	15 (31.9)	32 (68.1)
Spirulina	43	3 (7.0)	10 (23.3)	30 (69.7)
Protein powder†	28	-	6 (21.4)	22 (78.6)
Herb	25	2 (8.0)	4 (16.0)	19 (76.0)
Royal jelly	20	4 (20.0)	5 (25.0)	11 (55.0)
Chlorella	17	-	9 (52.9)	8 (47.0)
Immune Booster†	17	-	6 (35.3)	11 (64.7)
Lingzhi	16	-	2 (12.5)	14 (88.0)
Bird nest	16	6 (37.5)	9 (56.2)	1 (6.2)
Ginseng	14	-	-	14 (88.0)
Grape seed extract†	14	-	2 (14.3)	12 (85.7)
Mangosteen juice†	13	-	6 (46.2)	7 (53.8)
Porcupine date†	11	-	8 (72.7)	3 (27.3)
Noni juice†	10	-	4 (40.0)	6 (60.6)
Aloe vera†	10	-	4 (40.0)	6 (60.0)
Shark cartilage	7	-	-	7 (100.0)
Enzyme†	6	-	2 (33.3)	4 (66.7)
Jamu	1	-	-	1 (100.0)
*Traditional Medicine N = 129*				
Traditional Malay/indigenous medicine	96	8 (8.3)	12 (12.5)	76 (79.2)
Traditional Chinese medicine	24	-	5 (20.8)	19 (79.1)
Homeopathy	11	-	4	7 (63.6)

†Additional NPs via open-ended questions.

### Perceived helpfulness of CAM

Of all of the CAMs frequently used by >10% of the respondents, a total of 297 (95.9%) of the patients who performed PFH perceived that it was helpful. One hundred and thirty-two (78.6%) patients perceived that the second most frequently used CAM (i.e., consumption of vitamins and mineral supplements) was helpful. The third most frequently used CAM, the traditional healer, was perceived to be helpful by 76 of 96 (79.2%) of the patients who used it. Although fewer patients reported using relaxation exercises, massage, and meditation, compared with vitamin and mineral supplements, these MBPs were perceived to be more helpful by their users, compared with the users of vitamin and mineral supplements. Although <10% of the CAM users practiced tai chi and cupping, or consumed ginseng, shark cartilage or jamu, all of them perceived that these methods were helpful. In contrast, of the 16 patients who consumed bird’s nest, only one (6.2%) patient perceived that it was helpful. Of the four patients who opted for acupuncture, only two (50.0%) patients perceived that it was helpful. The results for the perceived helpfulness of CAM use are presented in Table 4.

### Reasons for using CAM

**Figure 3:**
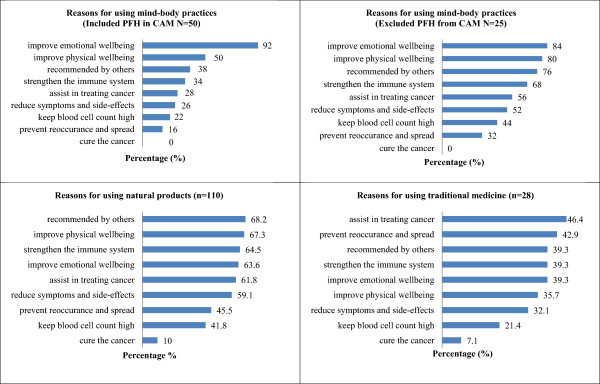
**Reasons for CAM use among patients.**

The main reason for using MBPs was the perception that CAM use improves emotional well-being (92.0% when PFH was included in CAM vs. 84.0% when PFH was excluded from CAM). Patients used NPs because they were recommended by others (68.2%). The use of TM was perceived to be an effective cancer treatment (46.4%). A minority of NP users (10.0%) and TM users (7.1%) reported that they expected these interventions to cure their cancer (Figure 3).

## Discussion

The findings revealed that CAM use was prevalent among breast cancer patients during chemotherapy, irrespective of whether PFH was included in, or excluded from, the definition of CAM. Most of the CAM users had a tertiary education, higher household income, were at advanced stages of cancer and were less likely to comply with the chemotherapy schedule. The prevalence of CAM use was higher than the prevalence values reported by Nurfaizah [19] and Soraya [20] for patients in Malaysia. These two previous studies of populations of breast cancer survivors had included prayer in the definition of CAM. The high prevalence in our study might be explained by the fact that the patients were receiving chemotherapy. It was reasonable for them to use CAM to provide relief from cancer-chemotherapy-related side effects and symptoms. Patients with more education and a higher economic status may have been more likely to search for other therapies to cope with the disease and treatment effects [32]. Likewise, patients at advanced stages of cancer may have experienced higher stress and lower immunity. Thus, they may have been more likely to use CAM for stress reduction and for strengthening the immune system to fight the disease [33]. These findings are consistent with the finding of other studies. Tertiary education [3, 19, 26, 34, 35], higher household income [7, 3] and an advanced stage of cancer [31, 36] are significantly associated with the use of CAM. In contrast, however, Arthur et al. [37] reported that compared with non-CAM users, CAM users had more control of their treatment options and how they were going to manage their lives after diagnosis. The results of the present study indicated that CAM users were less likely to comply with the chemotherapy schedule.

Exclusion of PFH from the definition of CAM reduced the prevalence of overall CAM and MBP use. The most common CAM use category switched to NPs, followed by MBPs, and TM. This result may be associated with the observation that most of the CAM users used PFH, so deletion of PFH from the CAM had excluded them as CAM users. This finding confirms the findings of Tippens et al. [29], who reported that excluding PFH from the definition of CAM may dramatically decrease the percentage of CAM users. Correspondingly, exclusion of PFH from the definition of CAM significantly decreased the number of MBP users. This finding is consistent with that of Barnes [38], who reported that the use of MBPs by African Americans decreased from 68.3% to 14.7% when PFH was excluded from the definition. In concordance with the NCCAM definition of CAM as “a group of diverse medical and health care systems, practices and products that are not presently considered to be part of conventional medicine” [24], prayer has been included in much CAM research [28]. However, although prayer and CAM are often interrelated, they are not the same [39]. Prayer is a universal human activity. The *Oxford Dictionary* defines prayer as “a solemn request for help or expression of thanks addressed to God or an object of worship” [40].

The findings indicated that the most popular CAM was PFH, followed by use of vitamin and mineral supplements, a Malay traditional healer, cleansing detoxifying diets, antioxidant capsule/tablets, breathing exercises, and massage. The majority of the patients included in this study were Malays. All Malays in Malaysia are Muslim and are obligated to repetitively recite five times daily a prayer/affirmation to Allah (God) and to the prophet Mohammed (Qur’an). Therefore, prayer is an activity that Malay breast cancer patients are likely to continue to routinely perform during chemotherapy. While praying (doa), it is common for Malay patients to ask Allah (God) to restore their health. They may also perform additional prayers, such as the repentance prayer (solat Taubat), and the request prayer (solat Hajat). In this study, the high prevalence of PFH indicated that inclusion of prayer as an MBP intervention resulted in increased CAM use, particularly in specific ethnic and racial groups [29]. The present findings, while preliminary, have clearly indicated the differences in CAM use when PFH was included in and excluded from the definition of CAM.

A significant key finding was that over one-half of NP users ingested more than one NP during chemotherapy. The most popular NP was vitamins and mineral supplements, followed by cleansing/detoxifying diets, antioxidant capsules/tablets, cactus juice and Spirulina. The extensive availability of over-the-counter NPs, as publicised by the media [41], and direct sales products recommended by friends or relatives, may be a factor. Despite evidence from several studies, MBPs intended to induce a natural relaxation response were not as popular as NP. The findings of these studies indicate that non-pharmacological CAM is beneficial for the management of chemotherapy side effects [42, 43]. Use of a traditional healer, which was included in the “traditional Malay/indigenous medical practices” category, was the most commonly used CAM practice, after PFH and vitamin and mineral supplements. This interesting finding may be attributed to the fact that traditional healers, who are regarded as the “primitive doctors” of a society, have treated people for generations. This result was similar to the result of a qualitative study performed in Malaysia by Merriam and Mazanah [44].

Of all of the CAMs frequently used by >10% of the CAM-group users, MBPs were perceived to be more helpful compared with NPs and TM. Most of the participants who performed prayer perceived that it was helpful. However, the effect of spiritual or religious beliefs on treatments and outcomes remains controversial [45]. Prayer may invoke a relaxation response [46], which in turn may positively affect health and overall well-being. Therefore, it seems appropriate for nurses to encourage patients to pray to increase their sense of spiritual well-being and wholeness, even though patients may feel healed but not cured [47, 48]. The perceived greater helpfulness of MBPs compared with the use of NPs suggests that more patients perceived a benefit from MBPs compared with NPs. Massage and aromatherapy massage confer short-term benefits in terms of psychological well-being, but there is insufficient conclusive evidence regarding this relationship [49]. Lee et al. [50] reported that the effectiveness of the use of qigong during cancer treatment is not yet supported by evidence from rigorous clinical trials. Similarly, the evidence that supports tai chi as an effective CT for cancer is not conclusive [51]. Conversely, results of a recent study indicated that medical qigong improves cancer patients’ overall quality of life and mood status and reduces specific treatment side effects. Medical qigong also may produce long-term physical benefits via the reduction of inflammation [52]. The results of an extensive systematic review performed by Zainal et al. [53] indicated that mindfulness-based stress reduction has significant potential for the reduction of stress, depression and anxiety in breast cancer patients. Raghavendra et al. [54] surmised that the use of yoga may reduce stress. Many patients perceived the use of MBPs to be beneficial. This result suggests that MBPs should be recommended as a supportive therapy and incorporated into integrative care. Some of these MBPs are relatively simple to learn, inexpensive and can easily be integrated into daily life.

The perceived benefit of consumption of NPs concurrently with chemotherapy is debatable. Evidence from at least one study supports the benefits of antioxidant supplement use during treatment [55]. One finding of this study indicated that most of the patients who used the services of traditional healers perceived that visits were helpful. This finding indicates that the patients had strong faith that traditional healers have an important role in the healing process. However, according to Merriam and Mazanah [44], high rates of delayed or interrupted breast cancer treatment may partially be because of widespread visits to traditional healers. Therefore, it is important to emphasise that the visits to traditional healers should complement, not replace, conventional medical treatment. When a patient feels respected and accepted, the relationship between the patient and nurses improves, and compliance with conventional medical treatment increases. There is a critical need for cancer awareness and education programmes that position traditional healers as complementary to conventional medicine approaches [44].

The main reason for using MBPs was the perception that MBP use improves emotional well-being. The reason for NP use was that it was recommended by others, while the use of TM was perceived to be able to treat cancer. This result suggests that many patients seemed to base their beliefs about the efficacy of ingested NPs on anecdotal evidence. This evidence is often based on personal experiences or opinions rather than objective controlled research studies. The use of CAM is not covered by the healthcare insurance systems in Malaysia [25], so this enthusiasm towards CAM suggests that many breast cancer patients were fairly optimistic about the use of CAM during chemotherapy.

### Implication for practice

NPs are often presented to be safer than conventional medicine approaches, but this is not necessarily correct. It is vital for nurses to guide patients in distinguishing between quackery and evidenced-based NPs, and to discern between complementary and alternative therapies. The findings that patients reported using NPs and TM to cure their cancer should be interpreted with caution. The results of a study performed by Al-Naggar et al. [26] indicate that 16.4% of cancer patients in Malaysia stop standard treatment while using CAM. This finding has serious implications because treatment choices can affect patient outcome. Patients should receive information about misconceptions about the use of traditional healers, which has not been shown to cure cancer. The practice guidelines of the Society for Integrative Oncology [56] recommend that unproven CAMs should not be used in place of conventional treatment, because delayed cancer treatment reduces the likelihood of remission or cure. It is imperative for nurses to familiarise themselves with the various CAMs that are most often used by patients during chemotherapy. They will then be able to answer patients’ questions about CAM use and be able to guide their patients as they seek additional information about, or referrals for, a particular therapy.

This study has several limitations. First, the setting was confined to two chemotherapy day-care centres, and the sample was mostly limited to urban women. Second, patients were recruited at a single point in time during different chemotherapy cycles. It was possible to compare CAM use between cycles, but we could not investigate trends during the six cycles of chemotherapy. A longitudinal study that prospectively follows up on patients over the entire course of chemotherapy should be performed to detect possible changes in CAM use across different treatment modalities.

## Conclusion

CAM use was prevalent among breast cancer patients, particularly among patients with higher education levels, higher household incomes and with advanced-stage cancer. These patients were also more likely to have poorer compliance with the chemotherapy schedule. The exclusion of prayer from the definition of CAM reduced the prevalence of overall CAM use. Many patients perceived that the use of MBPs was beneficial. This result suggests that MBPs should be recommended as supportive therapy.

## Electronic supplementary material

Additional file 1: **Questionnaire of CAM use.** (DOCX 42 KB)
