# Supplementary material for: Prayer-for-health and complementary alternative medicine use among Malaysian breast cancer patients during chemotherapy
Source: BMC Complement Altern Med. 2014 Oct 30;14:425. doi: 10.1186/1472-6882-14-425 (PMC4230750; doi:10.1186/1472-6882-14-425)
Supplement: Supplementary file 1 — Additional file 1: Questionnaire of CAM use. (DOCX 42 KB) [file 12906_2014_1999_MOESM1_ESM.docx]

CAM use questionnaire

|  | Part I Demographic, socioeconomic, disease and treatment characteristics | |
| --- | --- | --- |
|  | Age |  |
|  | Ethnic group | □Malay □Chinese □Indian □ Others |
|  | Educational level | □Primary/ lower □Secondary □Tertiary |
|  | Marital status | □Single □Ever married |
|  | Household income/month | □RM 3000 or less □More than RM 3000 |
|  | Staging of disease | □Early □Advanced |
|  | Menopausal status | □Pre menopause □Post menopause |
|  | Chemotherapy regime | □Docetaxel □FEC/ FAC/CML |
|  | Chemotherapy cycle | □2,3,4 □5,6 |
|  | Chemotherapy adherence | □Postponed □ on schedule |
|  | Part II CAM Usage | |
|  | Question: Which of the following CAM you may just started to use (at least 4 times) while you are undergoing chemotherapy?  □ Yes □No  Question: What is the perceived helpfulness of CAM that you have used?  □ 0 not applicable □1 not at all helpful □2 somewhat not helpful  □3 neither □4 somewhat helpful □5 very helpful | |
|  | Mind-body practices Perceived helpfulness | |
|  | Acupuncture □ Yes □No □ 0 □1 □2 □3 □4 □5 | |
|  | Aromatherapy □ Yes □No □ 0 □1 □2 □3 □4 □5 | |
|  | Cupping / *bekam* □ Yes □No □ 0 □1 □2 □3 □4 □5 | |
|  | Massage □ Yes □No □ 0 □1 □2 □3 □4 □5 | |
|  | Meditation □ Yes □No □ 0 □1 □2 □3 □4 □5 | |
|  | Prayer-for-health □ Yes □No □ 0 □1 □2 □3 □4 □5 | |
|  | Reiki □ Yes □No □ 0 □1 □2 □3 □4 □5 | |
|  | Structured exercise □ Yes □No □ 0 □1 □2 □3 □4 □5 | |
|  | Tai chi □ Yes □No □ 0 □1 □2 □3 □4 □5 | |
|  | Therapeutic /healing touch □ Yes □No □ 0 □1 □2 □3 □4 □5 | |
|  | Yoga □ Yes □No □ 0 □1 □2 □3 □4 □5 | |
|  | Others : □ 0 □1 □2 □3 □4 □5  □ 0 □1 □2 □3 □4 □5 | |
|  | Natural product Perceived helpfulness | |
|  | Antioxidants capsule / tablet □ Yes □No □ 0 □1 □2 □3 □4 □5 | |
|  | Bird’s nest □ Yes □No □ 0 □1 □2 □3 □4 □5 | |
|  | Chorella □ Yes □No □ 0 □1 □2 □3 □4 □5 | |
|  | Cleansing / detoxifying diet □ Yes □No □ 0 □1 □2 □3 □4 □5 | |
|  | Ginseng □ Yes □No □ 0 □1 □2 □3 □4 □5 | |
|  | Jamu □ Yes □No □ 0 □1 □2 □3 □4 □5 | |
|  | Lingzhi □ Yes □No □ 0 □1 □2 □3 □4 □5 | |
|  | Royal jelly □ Yes □No □ 0 □1 □2 □3 □4 □5 | |
|  | Shark cartilage □ Yes □No □ 0 □1 □2 □3 □4 □5 | |
|  | Spirulina □ Yes □No □ 0 □1 □2 □3 □4 □5 | |
|  | Unknown herbal □ Yes □No □ 0 □1 □2 □3 □4 □5 | |
|  | Vitamins & mineral supplements □ Yes □No □ 0 □1 □2 □3 □4 □5 | |
|  | Others : □ 0 □1 □2 □3 □4 □5  □ 0 □1 □2 □3 □4 □5 | |
|  | Traditional medicine Perceived helpfulness | |
|  | Homeopathy □ Yes □No □ 0 □1 □2 □3 □4 □5 | |
|  | Traditional Chinese medicine □ Yes □No □ 0 □1 □2 □3 □4 □5  ( Sinseh ) | |
|  | Traditional Indian medicine □ Yes □No □ 0 □1 □2 □3 □4 □5  (Ayurvedic / Siddha/ Unani) | |
|  | Traditional Malay/indigenous medicine □ 0 □1 □2 □3 □4 □5  ( Traditional healer) □ Yes □No | |
|  | Others : □ 0 □1 □2 □3 □4 □5  □ 0 □1 □2 □3 □4 □5 | |
|  | Part III Reasons for using CAM | |
|  | Question: What do you think about the reasons for your CAM use? | |
|  |  | |
|  |  | |
|  |  | |
|  |  | |
|  |  | |
